# Implementation of prehospital emergency ultrasound: the Sassari Prehospital Emergency Ultrasound Project

**DOI:** 10.1186/2036-7902-6-S2-A4

**Published:** 2014-08-27

**Authors:** D Mura, M Montomoli, D Masellis, P Delogu

**Affiliations:** 1 Sassari EMS, Siena EMS-Center for Simulation Training, Italy

## Background

Prehospital Emergency Ultrasound (PEU) is a growing field all over the world. Implementing point of care ultrasound (US) is a complex yet exciting task. We describe a new experience in the northern part of Sardinia Region, Italy, the Sassari Prehospital Emergency Ultrasound Project (SA-PEU Project).

## Aim of the study

To evaluate if a one day seminar and the collaboration with an expert center is a good strategy of PEU implementation.

## Methods

A partnership was established with an EMS yet successful in implementing PEU ( Siena EMS, Italy , which started the Siena PEU Project in 2009). The Sassari EMS, a physician-staffed service, acquired six US machines fit for the prehospital setting (Micromaxx/Sonosite). A motivational theoretical and hands-on simulated case-based seminar was conducted, involving all physicians working for the EMS. Self-tutorial videos were given and a plan for continuous training was developed. Then a questionnaire about clinical impact of the PEU and the appreciation of learning was administered (1- very poor to 5- very good).

## Results

50 ALS team leaders attended the 8 hours seminar and everyone understood the usefulness of focused US in solving a simulated EMS critical care scenario. The image acquisition was directly performed by each participant, showing that the technique could be mastered with a reasonable time devoted to training in the next months. Results from the questionnaire were (% is 4 or 5 evaluation): participants positively evaluated the seminar (94%) and the collaboration with an experienced EMS center (90%), considered the PEU strategy applicable in their usual setting of work (86%), useful in the management of emergency/urgency patients (82%), acquired enough skill to use PEU immediately (84%) .

## Conclusions

Our experience show that PEU strategy is a very helpful approach, and even a one-day seminar is an advantageous start in the implementation program. Benchmarking is a strong strategy when implementing such a novelty as PEU allowing the speediest spread of the methodology and limiting planning errors. A straightforward focus on the practical application of PEU at the first training appear to be useful in reducing the rate of “late adopters”. The project is ongoing to evaluate clinical outcomes.

